# Direct oral anticoagulant interference and removal in the factor VIII inhibitor assay

**DOI:** 10.1016/j.rpth.2025.103334

**Published:** 2025-12-29

**Authors:** Rick Timmerije, Saskia E.M. Schols, Daniëlle Meijer, Wideke Barteling, An K. Stroobants, Sanna R. Rijpma

**Affiliations:** 1 Laboratory of Hematology, Department of Laboratory Medicine, Radboud University Medical Center, Nijmegen, the Netherlands; 2 Synapse Research Institute, Maastricht, the Netherlands; 3 Hemophilia Treatment Center Nijmegen-Eindhoven-Maastricht, Nijmegen, the Netherlands; 4 Department of Hematology, Radboud University Medical Center, Nijmegen, the Netherlands; 5 Radboudumc Laboratory for Diagnostics, Department of Laboratory Medicine, Radboud University Medical Center, Nijmegen, the Netherlands

**Keywords:** DOAC measurement, DOAC removal, factor VIII, factor VIII inhibitor, inhibitor, interference

## Abstract

**Background:**

Direct oral anticoagulants (DOACs) interfere with clot-based assays, including factor (F)VIII testing and the Nijmegen–Bethesda assay, potentially leading to false-positive results for FVIII inhibitors. Misinterpretation of these results carries serious clinical consequences. Activated charcoal-based products, such as DOAC Remove, may restore assay accuracy, but data supporting their use in FVIII inhibitor assays are limited.

**Objectives:**

In this study, we aim to determine DOAC interference in FVIII inhibitor testing and evaluate effectivity of DOAC removal to restore assay reliability.

**Methods:**

Normal pooled plasma was spiked with therapeutic and supratherapeutic concentrations of apixaban, edoxaban, rivaroxaban, and dabigatran. Plasma pools from persons with acquired hemophilia A were spiked with DOACs and retested with and without DOAC removal treatment to evaluate workflow performance. FVIII inhibitor activity was measured with and without activated charcoal-based DOAC removal to establish DOAC interference and removal efficacy.

**Results:**

Spiking experiments with DOACs caused concentration-dependent false-positive FVIII inhibitor results (>0.6 Nijmegen Bethesda Units/mL), with dabigatran showing the strongest interference and apixaban the weakest. DOAC removal substantially reduced or eliminated interference across all drugs. Post-removal FVIII inhibitor values closely matched baseline concentrations in both spiked normal pooled plasma and pooled patient plasma, with most results within ±20% of the assay variability.

**Conclusion:**

DOACs significantly interfere with FVIII inhibitor assays, potentially causing clinically significant false-positive results. Activated charcoal-based DOAC removal treatment provides a practical solution to restore assay reliability. These findings support the integration of DOAC removal into anti-FVIII testing protocols for patients on anticoagulant therapy.

## Essentials

• DOACs may interfere with FVIII inhibitor assays.
• Interference with the FVIII inhibitor assay was evaluated for 4 DOACs. The effectiveness of DOAC removal was tested.
• All DOACs showed clinically relevant interference; DOAC removal treatment restored assay validity.
• Accurate FVIII inhibitor assessment is feasible with the correct approach toward DOAC interference.

## 1 Introduction

Direct oral anticoagulants (DOACs) are widely used to prevent and treat thromboembolic disease and to prevent thrombotic events [1]. Their increased use has highlighted challenges in laboratory testing, as many DOACs interfere with various *in vitro* hemostasis assays, complicating interpretation and potentially leading to clinical mismanagement [[2], [3], [4], [5]].

One of the vulnerable assays is the factor (F)VIII assay [6]. Similarly, the Nijmegen–Bethesda assay, which is based on the FVIII assay and the gold standard for quantifying FVIII inhibitors in persons with congenital or acquired hemophilia A (AHA), may be affected. False results of inhibitor presence or titer carry serious clinical implications. Overestimation may prompt unnecessary immunosuppression or bypass therapy, whereas attributing inhibitor presence to DOAC interference may disregard the possibility of actual inhibitor presence; therefore, abolished or delayed treatment could increase the risk of bleeding [7,8]. Activated charcoal-based products, such as DOAC Remove (5-Diagnostics), can efficiently adsorb and eliminate DOACs from plasma samples [9,10]. While their utility in restoring assay accuracy has been demonstrated in many assays, evidence of their use in FVIII inhibitor assays is limited.

We therefore aimed to (1) quantify the extent of DOAC interference with FVIII inhibitor testing, (2) compare the effects of 4 different DOACs (rivaroxaban, edoxaban, dabigatran, and apixaban) on the inhibitor assay, and (3) evaluate whether DOAC removal normalizes assay performance in both spiked normal pooled plasma (NPP) and pooled AHA-derived plasmas.

## 2 Methods

### 2.1 Preparation of plasma samples

Blood collection was conducted in accordance with the Declaration of Helsinki, and all participants (patients and healthy controls) provided general consent for the further use of their biological material. The material was collected under Commissie Mensgebonden Onderzoek (ethical committy for human research) protocol number 2013-064 and was not subject to the Medical Research Involving Human Subjects Act. An NPP batch was created by combining 40 individual plasmas from healthy donors. Women using oral contraceptives were excluded from participation. The NPP was flash-frozen and stored at −80 °C.

NPP was spiked with apixaban (Selleck Chemicals; 150-800 ng/mL), edoxaban (Selleck Chemicals; 150-800 ng/mL), rivaroxaban (Selleck Chemicals; 150-800 ng/mL), or dabigatran (Selleck Chemicals; 250-1000 ng/mL). For DOAC removal, samples spiked with 500 ng/mL of each drug were treated with DOAC Remove (5-Diagnostics) according to the manufacturer’s instructions.

Plasma from persons with AHA, collected during routine diagnostics and not receiving interfering medications, was pooled into 3, 5, and 8 Nijmegen Bethesda Units (NBU)/mL. These pools were spiked with 500 ng/mL of each DOAC and tested with and without DOAC Remove.

### 2.2 Measurement of DOACs

Apixaban, edoxaban, and rivaroxaban were quantified on an ACL TOP 350 CTS analyzer (Werfen) using the chromogenic HemosIL Liquid Anti-Xa assay (Werfen) with HemosIL rivaroxaban and apixaban controls/calibrators (Werfen) and BIOPHEN edoxaban controls/calibrators (Hyphen Biomed). Dabigatran was measured using the diluted thrombin time assay on the same analyzer, with HemosIL dabigatran controls/calibrators (Werfen).

### 2.3 FVIII inhibitor measurement

FVIII inhibitor activity was measured using the Nijmegen–Bethesda assay as described before [11]. Plasma was heat-inactivated at 58 °C for 1.5 hours, mixed 1:1 with imidazole-buffered NPP, and incubated at 37 °C for 2 hours. FVIII activity was determined using a one-stage clotting assay on an ACL TOP 550 analyzer (Werfen) with HemosIL reagents (Werfen). Titers ≥0.6 NBU/mL were considered positive [12,13]. DOAC removal was deemed reliable when posttreatment inhibitor levels were within ±20% of baseline.

## 3 Results and Discussion

### 3.1 Interference of DOACs in the anti-FVIII assay

**Figure 1:**
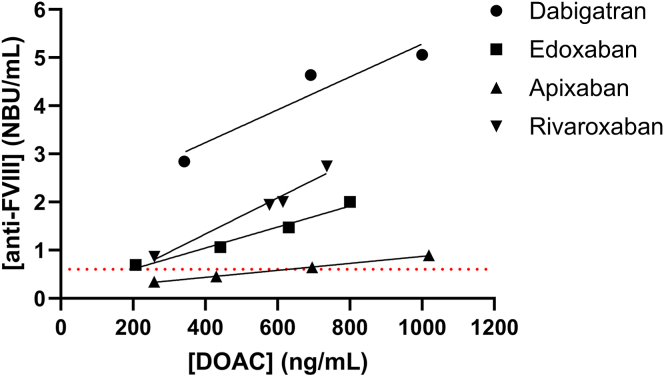
Interference of direct oral anticoagulants (DOACs) in the antifactor (F)VIII assay. Normal pooled plasma was spiked with DOACs at clinically relevant and supratherapeutic concentrations. The red dashed line indicates 0.6 Nijmegen Bethesda Units (NBU)/mL. A concentration-dependent increase in anti-FVIII levels (NBU/mL) was observed across all DOACs, with the strongest interference observed for dabigatran and the weakest for apixaban.

NPP spiked with therapeutic and supratherapeutic concentrations of dabigatran, edoxaban, rivaroxaban, and apixaban showed a concentration-dependent increase in FVIII inhibitor activity (Figure 1) [14]. All agents produced false-positive results (≥0.6 NBU/mL), except apixaban, which was <430 ng/mL. Dabigatran generated the strongest interference, whereas apixaban had the least effect on the test result.

### 3.2 Efficacy of DOAC removal

Treatment with DOAC Remove effectively reduced or eliminated all DOACs (Table). Dabigatran and rivaroxaban were fully cleared, and FVIII inhibitor activity was normalized in all cases. Edoxaban and apixaban were only partially removed, but residual levels did not cause clinically significant interference; posttreatment FVIII inhibitor titers were <0.6 NBU/mL in all samples.

**Table:** Direct oral anticoagulant concentrations and antifactor VIII measurements with and without direct oral anticoagulant removal.

DOAC	DOAC concentration (ng/mL)	DOAC concentration (ng/mL) + DR	Anti-FVIII (NBU/mL)	Anti-FVIII (NBU/mL) + DR
Dabigatran	1000^a^	0	5.1	0.1
	691	0	4.6	0.0
	341	0	2.8	0.1
Edoxaban	800	9	2.0	0.1
	630	7	1.5	0.2
	442	7	1.1	0.2
	207	11	0.7	0.3
Apixaban	1019	101	0.9	0.2
	695	101	0.6	0.2
	430	84	0.5	0.2
	258	67	0.3	0.0
Rivaroxaban	736	0	2.7	0.2
	615	0	2.0	0.2
	578	0	1.9	0.2
	259	0	0.9	0.1

DOAC, direct oral anticoagulant; DR, direct oral anticoagulant removal; FVIII, factor VIII; NBU, Nijmegen Bethesda Units.

a Could not be accurately determined due to assay range.

### 3.3 Validation of plasmas from persons with AHA

**Figure 2:**
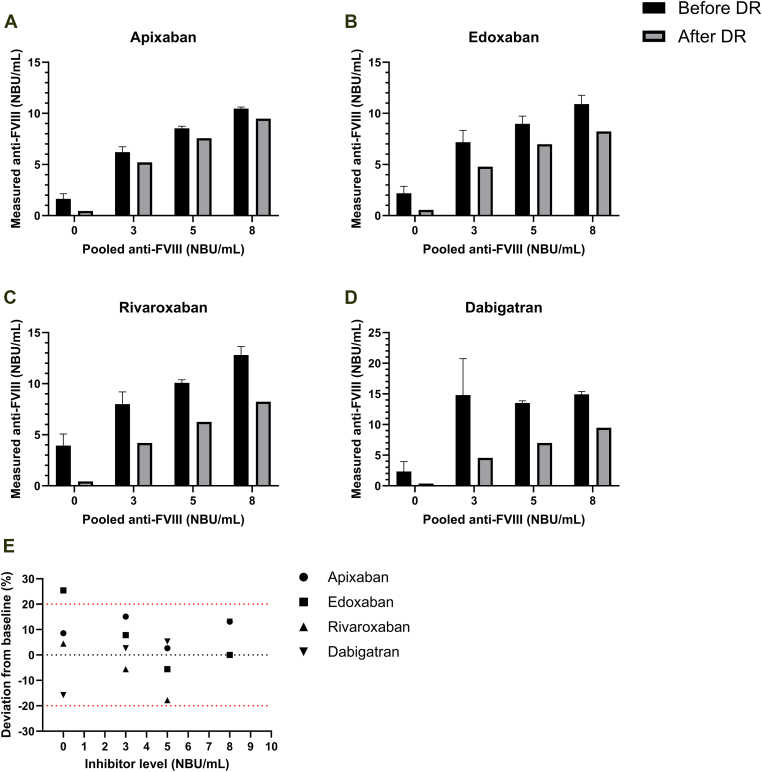
(A–D) Effect of direct oral anticoagulant (DOAC) removal (DR) on patient plasma pools. Plasma samples were pooled at increasing concentrations of antifactor (F)VIII (0-8 Nijmegen Bethesda Units [NBU]/mL) in the presence of 500 ng/mL of each DOAC and measured using the anti-FVIII assay. Anti-FVIII levels were measured before (*n* = 2) and after (*n* = 1) DR treatment. (E) Agreement between baseline and DR measurements across pooled anti-FVIII levels. Deviation was calculated for anti-FVIII levels measured after DR compared with baseline samples (without DOACs) at increasing anti-FVIII concentrations (0-8 NBU/mL). The red dashed line indicates a 20% deviation from the baseline measurement.

AHA plasma pools (0, 3, 5, and 8 NBU/mL) spiked with 500 ng/mL of each DOAC showed overestimation of inhibitor titers, which was the strongest for dabigatran and the least for apixaban. Following DOAC removal, FVIII inhibitor levels closely matched baseline values across all titers (Figure 2A–D). Nearly all postremoval results fell within ±20% deviation from the baseline (Figure 2E). The only exception was a slight variability in the edoxaban negative control, which remained well below the positive threshold.

### 3.4 Clinical and methodological implications

Misdiagnosis of FVIII inhibitors can have serious consequences. Indeed, there are 2 case reports that describe the delayed diagnosis of acquired hemophilia with inhibitors due to the use of a DOAC [15,16]. In acquired hemophilia, a false-positive result may lead to unnecessary initiation or prolonged use of immunosuppression, whereas in congenital hemophilia, it may result in inappropriate continuation of immune tolerance induction therapy or the unnecessary use of costly bypassing agents [8]. Fortunately, the clinical implications are limited. Apixaban, the most widely used DOAC, shows the least interference with FVIII inhibitor testing, whereas dabigatran, the least used DOAC, shows the strongest overestimation of FVIII inhibitor results [17].

Several approaches have been proposed to reduce DOAC interference in laboratory assays, including timing of blood draws to trough levels, using DOAC-insensitive reagents, or employing removal agents. Our data support the use of activated charcoal-based removal treatment as a robust and practical solution for the anti-FVIII assay, restoring accuracy in both spiked NPP and patient-derived samples.

### 3.5 Limitations and future directions

This study used pooled plasma samples and a single assay platform, which may not capture interindividual variability or interlaboratory differences. Even on a single platform, variation in the assay in some cases introduces uncertainty in the effect size of DOAC interference in anti-FVIII assays. For example, 500 ng/mL dabigatran was spiked into a 3 NBU/mL sample (Figure 2D). DOAC concentrations were selected to cover the therapeutic and supratherapeutic ranges but may not represent all clinical scenarios. Furthermore, the effectiveness of alternative DOAC removal agents has not been evaluated; these may be more effective in removing apixaban and, consequently, its interference. Nevertheless, the results were consistent across agents and experimental conditions, supporting the generalizability of the findings.

Larger multicenter studies are needed to validate these results across different assay platforms and to establish standardized protocols for DOAC removal in inhibitor testing. Further evaluation of FIX inhibitor assays is also warranted, given the likelihood of similar interference; however, the occurrence of FIX inhibitors against FIX is rare.

## 4 Conclusion

This study demonstrates that the DOACs apixaban, edoxaban, rivaroxaban, and dabigatran can cause concentration-dependent false-positive results in the FVIII inhibitor assay. Additionally, we showed that activated charcoal-based DOAC removal treatment effectively reduced or eliminated this interference, restoring reliable inhibitor quantification even in samples with high DOAC concentrations. These findings support the routine implementation of DOAC removal protocols to improve diagnostic accuracy and patient safety in inhibitor assays.
